# ECG Marker Evaluation for the Machine-Learning-Based Classification of Acute and Chronic Phases of *Trypanosoma cruzi* Infection in a Murine Model

**DOI:** 10.3390/tropicalmed8030157

**Published:** 2023-03-04

**Authors:** Paulina Haro, Nidiyare Hevia-Montiel, Jorge Perez-Gonzalez

**Affiliations:** 1 Instituto de Investigaciones en Ciencias Veterinarias, Universidad Autónoma de Baja California, Mexicali 21386, Baja California, Mexico; 2 Unidad Académica del Instituto de Investigaciones en Matemáticas Aplicadas y en Sistemas del Estado de Yucatán, Universidad Nacional Autónoma de México, Sierra Papacal 97302, Yucatan, Mexico

**Keywords:** automatic classification, Chagas disease, feature selection, machine learning, *Trypanosoma cruzi*

## Abstract

Chagas disease (CD) is a neglected parasitic disease caused by the protozoan *Trypanosoma cruzi* (*T. cruzi*). The disease has two clinical phases: acute and chronic. In the acute phase, the parasite circulates in the blood. The infection can be asymptomatic or can cause unspecific clinical symptoms. During the chronic phase, the infection can cause electrical conduction abnormalities and progress to cardiac failure. The use of an electrocardiogram (ECG) has been a methodology for diagnosing and monitoring CD, but it is necessary to study the ECG signals to better understand the behavior of the disease. The aim of this study is to analyze different ECG markers using machine-learning-based algorithms for the classification of the acute and chronic phases of *T. cruzi* infection in a murine experimental model. The presented methodology includes a statistical analysis of control vs. infected models in both phases, followed by an automatic selection of ECG descriptors and the implementation of several machine learning algorithms for the automatic classification of control vs. infected mice in acute and/or chronic phases (binomial classification), as well as a multiclass classification strategy (control vs. the acute group vs. the chronic group). Feature selection analysis showed that P wave duration, R and P wave voltages, and the QRS complex are some of the most important descriptors. The classifiers showed good results in detecting the acute phase of infection (with an accuracy of 87.5%), as well as in multiclass classification (control vs. the acute group vs. the chronic group), with an accuracy of 91.3%. These results suggest that it is possible to detect infection at different phases, which can help in experimental and clinical studies of CD.

## 1. Introduction

Chagas disease (CD) or American trypanosomiasis is a neglected parasitic disease caused by the protozoan *Trypanosoma cruzi* (Chagas, 1909) (Kinetoplastida, Trypanosomatidae) (*T. cruzi*). It affects 6–7 million people worldwide, and most of the cases occur in Latin America [1,2]. *Trypanosoma cruzi* has a tropism for muscle cells, including cardiac muscle cells. Myocardium damage is caused by different pathophysiological mechanisms, such as autoimmunity, inflammatory immune response, direct cardiomyocyte damage, nerve damage, and damage caused by vascular anomalies that can induce fibrosis, ischemia, arrhythmias, and thrombosis, triggering progressive heart failure and death [3].

The clinical diagnosis of CD is a challenge. The disease has two different phases: acute and chronic. The acute phase, lasting 4–8 weeks, is asymptomatic in most cases, and when signs are present, they are usually nonspecific [4]. Because of that, the clinical detection of the infection in early phases in humans is rarely achieved and still poorly understood. For its diagnosis, a parasitic test (a search for evidence of the parasite circulating in the bloodstream) is performed, but no antibody titers are determined due to their appearance 3–4 weeks after infection. The acute phase responds better to treatment, and a cure can be achieved at this phase [5], hence the importance of an early detection of the infection. The chronic phase in humans has an asymptomatic phase (possibly lasting 10–30 years), and after a period of time, an estimated 30–40% of infected cases can transit to the symptomatic phase. The asymptomatic phase is usually diagnosed by finding antibodies of *T. cruzi* in a blood test [4].

An electrocardiogram (ECG) is a relevant non-invasive diagnostic tool for studies of electrical heart activity, which is widely used in the diagnosis and follow-up of patients. During the chronic phase, the presence of ECG abnormalities determines the start of the symptomatic CD. The ECG is one of the most important diagnostic techniques, second only to the positive parasitic or antibody test. This is because ECG signal changes are prevalent in both children and adults infected with *T. cruzi* [6]. The ECG abnormalities associated with the presence of infection in positive infected humans compared to a non-positive population are affections of conduction at the ventricular level, such as a complete right bundle branch block (RBBB) and a left anterior fascicular block (LAFB), separately or in combination [6,7]. Nodal-type conduction abnormalities, such as a first-degree atrioventricular block (AV-B) and atrial flutter (AF), occur as well [6], although the latter is not consistent with all studies.

The use of an ECG to study the cardiomyopathy caused by *T. cruzi* infection in a murine model has been helpful for better understanding the infection phases and disease pathophysiology, exploring the effect of new drugs and their combinations, and vaccine development. The murine model allows us to recreate acute and chronic infection phases within a short period of time. The electrocardiographic trace can be obtained by telemetry, under anesthesia, and in conscious animals, depending on the equipment available. Heart rate and other parameters related to the interval duration and segment trace are usually recorded. It is not usual to obtain information about voltage during these studies, since it is required that the animal has contact with at least three electrodes, which is not possible with some equipment for conscious animals. The main findings in infected animals are arrhythmia development and second-degree AV blocks (AVB2 and AVB1), an increase in the P wave and QT complex, a prolonged PR interval, and the QRST complex, although fibrillation is a rare finding [8]. Campos et al. [9] report bradycardia, an AV block, and increased QT and PR intervals as significant alterations caused by *T. cruzi* infection in this model. Navarro et al. [10] found an association between prolonged QTc interval durations and a higher parasite load during the acute phase of infection.

The main objective of this work is to evaluate the contribution of different ECG components for the machine learning algorithm-based classification of control vs. *T. cruzi* infected mice models at different infection phases (the acute and chronic phase).

Several authors have recently implemented machine learning algorithms for the study of ECG descriptors and/or infectious diseases [11,12,13,14]. Regarding CD disease, Silva et al. [15] proposed that Heart Rate Variability (HRV) indices can be used to predict morpho-functional parameters from ECGs in infected patients. They implemented Random Forest (RF), Artificial Neural Networks (ANN), K-Nearest Neighbors (KNN), and Support Vector Machine (SVM) classifiers by using HRV indices. Silva et al. [15] showed that HRV indices are associated with cardiac morpho-functional properties in patients with CD. Vizcardo et al. proposed computational strategies for the study of different ECG variables in patients with CD. These authors have proposed the analysis of HRV indices in both temporal and frequency domains [16], and they have proposed the approximate entropy and sample entropy implementation, together with a logistic regression classifier, as well as the Kruskal–Wallis test to differentiate between control and CD patients [17,18,19]. They have also proposed the principal component analysis of average circadian profiles together with HRV indices [20]. In a previous work [21], the intervals and durations of some ECG complexes were analyzed in order to integrate them into a multimodal automatic classification strategy. However, in that work, the voltages and the characteristics of the P, J, and T waves were not analyzed. In general, the previously described works presented classification strategies and analyses between a control group and a single phase of infection (acute or chronic). Additionally, most of the reported works do not consider voltage-derived descriptors in their analysis.

In contrast to previously reported works, in this study we present an automatic ECG descriptor selection approach for multiclass classification considering different phases of *T. cruzi* infection. In addition, voltage markers for the study of infection have been included in the proposed analysis. The main contributions of this work are (1) a statistical analysis of ECG descriptors (e.g., duration, voltage, intervals, segments, complexes, and characteristics) in acute and chronic infected mice vs. matched controls of each phase, (2) a strategy for estimating the ECG variables—contribution and selection—that help to discriminate between infected groups (acute and/or chronic phases) vs. controls, and (3) the implementation of machine learning algorithms for automatic binary and multiclass classification using ECG descriptors for infected mice in both phases (acute and/or chronic) vs. control mice.

## 2. Materials & Methods

### 2.1. A Murine Model Description for T. cruzi Infection

A total of 181 healthy female ICR (Albino mice group from the Institute of Cancer Research) mice, aged 6–8 weeks, were employed. The animals were handled according to the *Care and Use of Laboratory Animals Guide* (eighth edition). The protocol was approved by the Ethics Committee of the Centro de Investigaciones Regionales Dr. Hideyo Noguchi (CIRB-006-2017) at the Universidad Autónoma de Yucatán, México. The animals were housed in polycarbonate cages (39.6 × 21.2 × 17.2 cm), where shaving was used as a bed. General conditions were controlled: a temperature from 19 to 24 °C and a humidity of 70%, with light for 12 h and darkness for 12 h. Commercial feed and water were provided ad libitum. Cage cleaning and changing were carried out every three days.

For the experimental study of the acute phase, a total of 96 animals were studied (66 infected and 30 controls). For the chronic phase, a total of 85 models were analyzed (61 infected and 24 controls). To differentiate between the two control groups, the group corresponding to the acute phase will be denoted as “Control 1”, and the group corresponding to the chronic phase will be named “Control 2”. For the acute phase, 66 mice were infected by inoculation with 1000 blood trypomastigotes of strain H1 (TcI lineage) *T. cruzi* via intraperitoneal (IP); in the chronic phase, 61 animals were inoculated with 500 blood trypomastigotes, using the same strain and administration. For control groups (1 and 2), animals were administered with a physiological saline solution IP.

Infection development was corroborated with parasitemia monitoring every 5 days at 5, 15, 20, 25, 30, and 35 days for the acute phase and every 30 days for the chronic phase until Day 120 after inoculation. Parasitemia was calculated by counting the *T. cruzi* parasites in the Neubauer chamber, where a blood sample of 10 µL was obtained from the tail of the mouse through a distal area cut. The parasites number per milliliter was calculated as

(1) $$\mathrm{Parasite}\mathrm{Number}(\mathrm{per}\mathrm{mL})=\frac{\mathrm{Parasites}\mathrm{Counted}}{4(\mathrm{Dilution}\mathrm{Factor})(\mathrm{Camera}\mathrm{Depth})}$$

### 2.2. ECG Data Acquisition

ECG signals were acquired at different days: (a) for the acute group and Control 1, the acquisitions were at Days 5, 15, 25, and 35 post-infection; (b) for the chronic group and Control 2, the acquisitions were at Days 60, 90, and 120 post-infection. To acquire ECG signals, the animals of all groups were anesthetized with isoflurane as inhaled anesthesia (Patterson scientific®, Waukesha, WI, USA). The animal was placed in an induction chamber at a dose of 3% and 0.5 L/min of O${}_{2}$. Once the animal was in a deep anesthetic plane, anesthesia was maintained with a mask at a dose of 1–2%. The animal was placed in a supine position with the forelimbs and hindlimbs fixed with tape to the ECG electrode platform (surgical monitor INDUS^®^, Webster, TX, USA). Additionally, a rectal probe was placed in order to monitor heart rate, respiratory rate, and body temperature throughout the process with the help of a rodent surgical monitor. ECG signals were obtained using derivative II and 0.2 mV; for each acquisition, a digital image with the ECG traces was obtained (these images were stored for later analysis). Subsequently, the animal was recovered from the anesthesia, the following day it was euthanized, and necropsy was performed. Finally, an expert manually measured several markers from the images of the ECG traces. For each mouse, three complexes were measured, and each descriptor was averaged.

**Figure 1:**
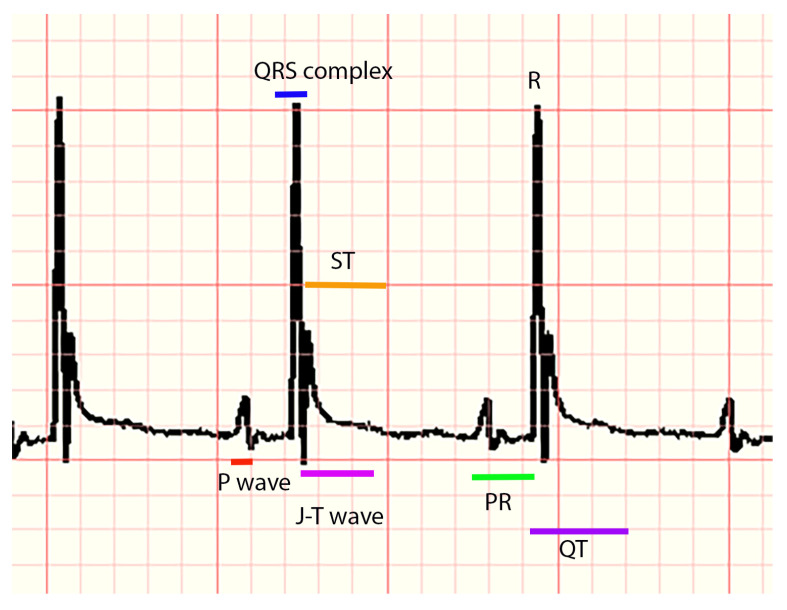
ECG of a healthy mouse. The P wave (atrial depolarization), followed by the QRS complex (ventricular depolarization), the R wave (depolarization of the ventricles), the J wave (early ventricular repolarization), the T wave (ventricular repolarization), the PR interval (time for electrical conduction from the atria to the ventricle), the ST segment (ventricular repolarization), and QT (ventricular depolarization repolarization).

The following parameters were measured for each animal, considering distances in milliseconds (ms) and voltage (amplitude) in millivolts (mV): P, Q, R, and S waves, the QRS complex, PR, QT, QTc intervals (the modified Bazett equation was used to obtain this interval), and the segments PR and ST. In addition, the parameters of heart rate and RR interval and the appearance of P, J, and T waves (presence, absence, positive, negative, or biphasic) were recorded (a representative ECG trace can be seen in Figure 1). All measurements were corroborated by another expert. For the analysis of ECG markers, statistical tests, and the implementation of machine learning algorithms, Python 3.8 was used on an Intel Core I7, 26 GHz computer with 16 GB of RAM.

### 2.3. Statistical Analysis

**Figure 2:**
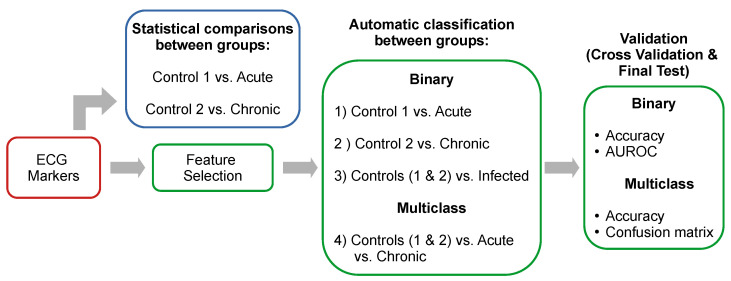
Proposed methodology for the analysis of ECG descriptors. In blue is the stage concerning statistical comparisons. The green section corresponds to the proposed methodology for feature selection, binary and multiclass classification, and the validation stage.

First, the acquired database was cleaned by discarding samples with empty data, atypical samples (data greater than ± 3 standard deviations), and data with errors at the time of capture, obtaining the final number of data analyzed for each phase (Table 1). As can be seen, the size of the classes in each study group is different; this is due to the mortality of some mice and to data discarded in database cleaning. Second, a statistical analysis of the main ECG components was carried out. This analysis consisted of a kurtosis test to check if the data present a normal distribution, followed by a Wilcoxon test without correction (since data shows a distribution non-Gaussian). The data comparison was (a) between Control 1 and the group with acute infection and (b) between Control 2 and the group with chronic infection. All analyses were performed pairwise by infection weeks, where two significance levels were considered p≤0.05 and p≤0.01. The proposed pipeline for ECG descriptors analysis (Figure 2) includes statistical analysis (Section 2.3), a feature selection stage (Section 2.4), and binary and multiclass classification of the different phases of infection (Section 2.5).

### 2.4. Feature Selection

To observe the contribution of the ECG components, an analysis of the importance of the features was performed using the Mean Decrease in Impurity (MDI) metric [22]. The MDI indicates the degree of impurity (classification capability) for each variable according to the GINI index, which measures the probability of a particular variable being wrongly classified when it is randomly chosen. This algorithm has been selected because of its ability to assign the importance of biological features [11,23,24]. Furthermore, in a previous work in which various feature selection algorithms were compared, the MDI-based approach was one of the best performing [21]. To calculate the MDI, the Extremely Randomized Trees Classifier algorithm was used with a total of 500 decision trees. The contribution of the ECG components was conducted for the following four study groups: (1) Control 1 vs. acute infection, (2) Control 2 vs. chronic infection, (3) Control 1 and 2 vs. infected (acute phase + chronic phase), and (4) Control 1 and 2 vs. acute infection vs. chronic infection. The feature selection methodology was implemented prior to cross-validation and final testing (as shown in Figure 2).

### 2.5. Automatic Classification and Validation

Three supervised classification algorithms were used: the Artificial Neural Network (ANN) [25], Random Forest (RF) [22], and Support Vector Machine (SVM) algorithms [26]. The ANN was trained using a ReLU (Rectified Linear Unit) activation function and stochastic gradient descent. The number of hidden layers and the L2 penalty were optimized. For the RF classifier, the number and depth of the tree, the impurity criterion (GINI/Entropy), and the number of features considered when seeking the best classification were optimized. Finally, the SVM was trained using a radial basis kernel, and the *c* and gamma hyperparameters were optimized. Classification algorithms were implemented for the four described groups: (1) Control 1 vs. the group with acute infection, (2) Control 2 vs. the group with chronic infection, (3) Control 1 and 2 vs. infected general group (acute phase + chronic phase), and (4) Control 1 and 2 vs. the group with acute infection vs. the group with chronic infection, as it is shown in Table 1.

The classifiers were fed with all the previously described ECG variables, as well as a subset of the most important variables according to the MDI obtained for each study group.

The four groups for automatic classification are shown in Table 1. In addition, the age range of studied mice in days, the number of total mice analyzed for each group, the data used for four-fold cross-validation (4-fold-CV), and the final test (FT) with unseen data are presented. In addition, to corroborate the consistency of the results obtained, an additional final test was performed with a synthetically augmented dataset (DA). Data augmentation is a technique widely used in various medical and biological applications with the objective of increasing sample size computationally [27]. To increase the dataset, the Synthetic Minority Over-Sampling Technique for Nominal and Continuous (SMOTE-NC) algorithm was implemented [28,29]. The penultimate column of Table 1 shows the number of increased data per class. These data were used for a second final test with unseen data.

Accuracy (ACC), the Area Under the ROC Curve (AUROC), and the Confusion Matrix (CM) were used to assess classification performance, where the ACC is the ratio of the number of well-classified data to the total amount of data, and the AUROC metric measures the posterior probability of each piece of classified data for a given class, i.e., a numeric value that represents the degree to which an instance is a member of a class. Both metrics (ACC and AUROC) range from 0 to 1, where 1 means a perfect classification. The CM is a matrix showing the true labels vs. the predicted labels; this matrix was only used to evaluate the multiclass classification, as can be seen in the last column of Table 1.

## 3. Results

During the acute phase, parasites circulating in blood were detected from Day 15 to Day 25–30 post-infection. In the case of the chronic phase, the parasite count was detectable only at Day 30, and no parasites were detectable during Day 60, 90, and 120.

**Figure 3:**
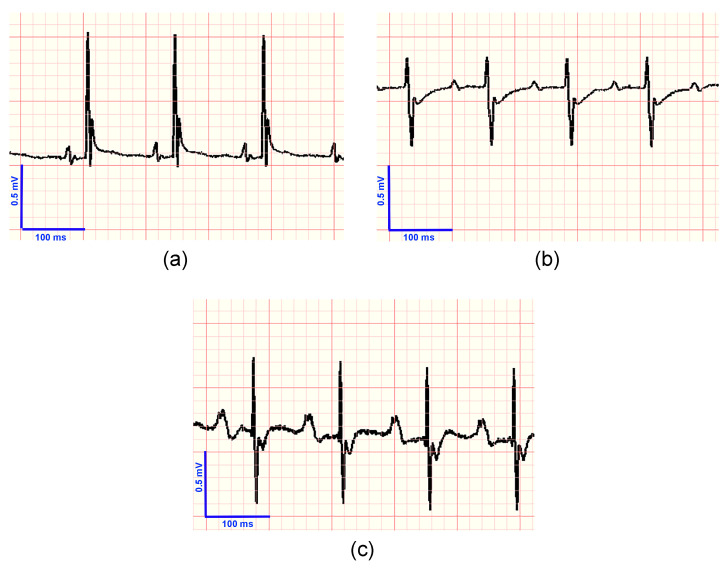
Examples of ECG images of a control mouse (**a**), and a mouse infected with *Trypanosoma cruzi* during acute (**b**) and chronic (**c**) phases. It can be seen that the most relevant findings are the prolongation of the PR interval, associated with a first degree atrioventricular blockade, a low voltage of R, which may reflect an inability of the myocardium to generate voltage due to miocarditis, and a negative T wave, which may indicate subepicardial ischemia or a bundle branch block. During the chronic phase, an increase in the voltage and duration of the P wave with a biphasic shape can be observed (**c**). This finding can be associated with an enlargement of the right and left atria. An increase in the duration of the PR interval can also be seen.

In Figure 3, the ECG traces for a control mouse (Figure 3a), a mouse in the acute phase of infection (Figure 3b), and a mouse in the chronic phase (Figure 3c) can be seen.

Statistical comparisons (Table 2) are shown below: (a) Control 1 vs. the mice with acute infection and (b) Control 2 vs. the group with chronic infection. Comparisons were made for each ECG descriptor by a Wilcoxon test without correction (statistical differences are shown in bold). It can be seen that the P wave voltage and the PR and QTc intervals show significant differences for both groups. In addition, the PR segment, the T wave duration, and the QT interval present differences for Control 1 vs. the acute groups. In the case of statistical comparison between Control 2 and the chronic groups, the heart rate, P wave duration, R and QRS voltages, and RR interval show statistical differences.

Regarding the comparison of Control 1 (N = 12) vs. the group with acute infection (N = 12), the P wave presented a positive complex for 12 controls and 9 mice with acute infection, as well as a biphasic wave for 3 mice from the acute group. The presence of the J wave was shown in 12 controls and 7 acute (5 mice with acute infection showed absence of this wave). For the T wave, 12 control and 6 acute mice presented a positive wave, and 6 acute mice presented a negative wave. In the comparison of Control 2 (N = 15) vs. the group with chronic infection (N = 15), for the P wave, a total of 15 control and 12 chronic mice showed a positive wave, and 3 mice with chronic infection presented a biphasic wave. The presence of the J wave was observed for all mice (15 controls and 15 chronic). For the T wave, 12 controls and 10 chronic mice showed a positive wave, and 3 controls and 5 chronic mice had a negative complex.

**Figure 4:**
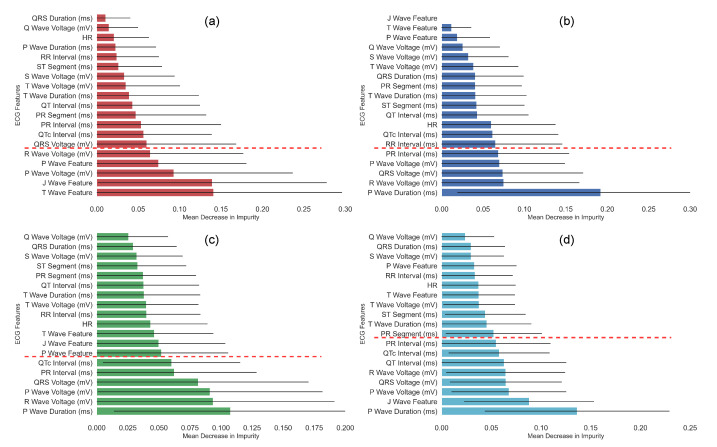
Analysis of ECG descriptors importance using the Mean Decrease in Impurity (MDI). (**a**) Comparison of Control 1 vs. the group with acute infection, (**b**) Control 2 vs. the group with chronic infection, (**c**) Control 1 and 2 vs. the infected groups (acute and chronic), and (**d**) Control 1 and 2 vs. the acute group vs. the chronic group (multiclass analysis). Each bar represents the mean value, the black lines the standard deviation, and the red dotted lines the threshold used to select the most relevant descriptors.

The results of the feature importance according to the MDI are presented in Figure 4. For all graphs, the bar shows an average MDI. The black line is the standard deviation obtained from 500 estimators, and the red dotted line indicates the threshold used to select the most relevant descriptors. As can be seen, the different phases of infection present changes in the significance of ECG markers. Comparison of Control 1 vs. the acute groups (Figure 4a) shows that the main ECG variables were characteristic of the T, J, and P waves, as was the voltage of the R and P waves. In contrast, the increase of the P wave and the PR interval time and changes in the voltage of the R and P waves and the QRS complex were the most relevant descriptors in the comparison of Control 2 vs. mice with chronic infection (Figure 4b). In the comparison of Control 1 and 2 vs. infected (Figure 4c), it can be seen that the selected variables were similar to the Control 2 vs. chronic group comparison, the most relevant descriptors being the voltage of the P and R waves and the QRS complex, as well as the P wave duration and the QTc and PR intervals. Regarding the multiclass classification of Control 1 and 2 vs. the acute group vs. the chronic group (Figure 4d), a combination can be noted between the most representative variables shown in the previous selections (Figure 4a–c), where the J wave characteristic, the P wave duration, while voltage of the P wave, R wave, and QRS complex are preserved. Unlike previous analyses, the QT interval was also shown to be relevant in the multiclass feature selection.

The results of the automatic classification using the RF, ANN, and SVM algorithms, with and without feature selection (FS), are shown in Table 3. The reported results are according to the ACC and AUROC obtained with four-fold cross-validation. In general, it can be seen that performance improves with the selection of features for the three proposed classifiers. The best performances were shown by the RF + FS classifier with an AUROC of 100% for Control 1 vs. acute group classification, an ACC of 87.5% and an AUROC of 100% for Control 2 vs. the chronic group, an ACC of 82.5% and an AUROC of 89% for the case of Control 1 and 2 vs. the infected group, and an ACC of 71.6% for the multiclass classification (Control 1 and 2 vs. the acute group vs. the chronic group).

The results for each classifier with and without feature selection for the final test with unseen data are shown in Table 4. It can be seen that the results obtained by the three classifiers are similar, except for the multiclass classification, where RF+FS shows the best performance with an ACC of 91.3% (last column). It can also be noted that the results during the final test are in the range of the results obtained during the cross-validation, except for the classification of Control 2 vs. the chronic group, with an ACC of 66.6%.

**Figure 5:**
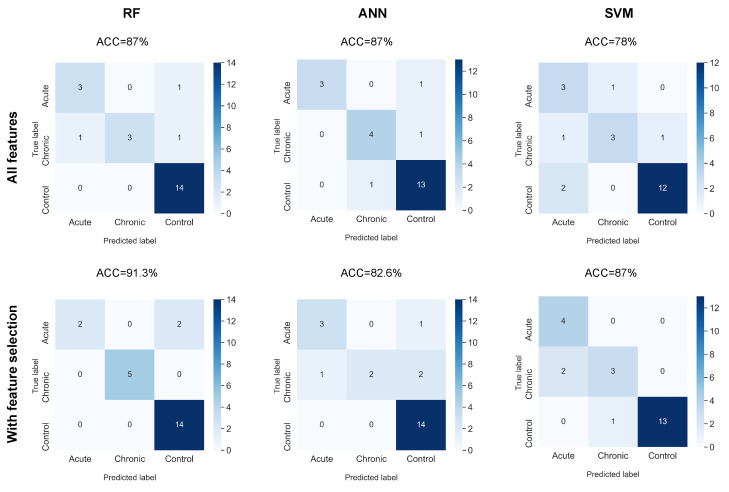
Multiclass classification confusion matrices (Control 1 and 2 vs. the acute group vs. the chronic group) for the final test with hold-out data.

The results of the final test of the multiclass classification using a confusion matrix are shown in Figure 5. It can be seen that the yields obtained exceed a 78% ACC, with the RF + FS combination showing the best performance with an ACC of 91.3%. For this combination of algorithms, it can be noted that only two mice with acute infection were misclassified as controls. This may be because acute infection is an early phase, and there may not be significant changes in ECG markers in the first few weeks of infection in some animals. In the case of SVM + FS, an improvement in performance was obtained, which is in agreement with that shown by RF + FS. However, for the case of ANN + FS, a decrease can be observed when including feature selection. When observing the confusion matrix in detail, it can be noted that the errors were in the incorrect classification of the chronic phase. The chronic phase is when there may be a greater variety in the alterations identified by the ECG. These changes may alter the ECG markers and therefore make classification by ANN more difficult.

Table 5 shows the final test results for each classifier using the augmented dataset (also unseen data) with and without feature selection. In general, it can be observed that the results presented are consistent with those shown in Table 4, with RF + FS followed by SVM + FS as the best performing classifiers. As previously described, the purpose of the evaluation with augmented data is to verify the consistency of the results obtained in the previous tests.

## 4. Discussion

The ICR mice group is a good model for the study of *T. cruzi* infection. Acute and chronic infection phases were established, and changes in ECG were determined in these two phases. ECG is the first test to be performed during the initial clinical evaluation of diagnosed CD patients after serology. It is also important to be able to determine the set of the most typical ECG findings for the identification of patients with CD infection with no diagnosis yet, as this could raise the suspicion of clinicians and add CD on the list of presumptive diagnoses. Particularly in patients from endemic regions, it can lead to further tests for the identification of infection by serology.

During *T. cruzi*, acute infection in mice changes on the T, J, and P waves were detected. Regarding the T wave, it appeared negative in half (6/12) of the infected cases, and the J wave was absent in five animals. The depolarization and repolarization of the ventricle is represented by T and J (early repolarization in mice is not present in humans). T wave inversion may indicate subepicardial ischemia, a bundle branch block, and/or right ventricle hypertrophy. The latter is less likely, due to the short time period of the *T. cruzi* infection course. The absence of J waves can be related to a delay in the ventricular repolarization [30]. A biphasic P wave was present in three mice (this wave represents the atrial depolarization). A biphasic wave can be caused by a rotation of the heart in the thoracic cavity or by left atrial enlargement. On the other hand, the finding of an increase in the voltage of the P wave can be a signal of an enlargement of the right atrium and/or lung disease such as pulmonary hypertension [30]. R deflection represents the depolarization of the ventricles. The decrease of the voltage of the R wave may reflect an inability of the myocardium to generate voltage due to inflammation of the cardiac muscle [30]. These are all possibly due to the extensive inflammation of the myocardium by the high numbers of parasites nesting in the cardiac muscle during this phase of infection.

Little is known about the early phases of CD cardiac dysfunction in humans. A murine model is helpful in understanding the early cardiac electrical pathophysiology of the disease. P wave abnormalities are not usually found in humans in CD or murine models of *T. cruzi* infection. The findings on the P wave possibly associated with early cardiac bilateral atrial enlargement and the ventricular depolarization and repolarization dysfuction related to R wave changes detected could be of importance for the diagnosis of early CD infection.

Infected individuals in the chronic phase can be asymptomatic or symptomatic. The asymptomatic phase can last 10–30 years. Patients are positive to the serologic test, but no ECG alterations are present. Most of the cases stay asymptomatic during life, but it is estimated that 30–40% of the infected humans transit to the symptomatic phase. During the chronic symptomatic phase, no parasites are circulating in the bloodstream; instead, parasites can form nests in the myocardium, and clinical signs are evident. The ECG helps to determine this transition by the detection of initial abnormalities [4]. In non-endemic territories or when the suspicion of CD is not yet established by the clinician, the ECG abnormalities described in this work can be helpful in the identification of these cases. Further studies on humans are needed.

During the chronic phase of *T. cruzi* infection in mice, the findings of an increase in the duration of the P wave may be related to an increase in the size of the left atrium. The PR interval represents the start of atrial and ventricular repolarization, and the delay found represents a first-degree atrioventricular blockade. The decrease in voltage of the R wave and QRS may reflect myocardial dysfunction due to myocarditis caused by inflammation, parasitic invasion, hypoxia, or myocytolysis [30]. With respect to Control 1 and 2 vs. infected, the selection of relevant descriptors was the same as those obtained in the chronic phase, adding the QTc interval (QTc is the corrected QT interval). Because QT (the beginning of the ventricular depolarization to repolarization) varies normally with heart rate, a corrected interval is used to assess the absolute QT prolongation. The finding of its reduction is associated with inflammation in the cardiac muscle caused by *T. cruzi* infection [31].

The damage occurring in early phases of infection due to the extensive inflammation that perpetuates at the chronic phase, causing fibrosis, ventricular hypertrophy, left atrial enlargement possibly associated with pulmonary hypertension, and a first grade AV blockade with ventricular depolarization dysfunction.

Regarding the multiclass analysis of Control 1 and 2 vs. the acute group vs. the chronic group, the most representative ECG descriptors were the same as those shown in the previous analyses, with the addition of the QT interval. This reflects that the combination of relevant variables found in the acute and chronic phases extend to the multiclass analysis of infection.

As described in the previous sections, the selection of the most important ECG descriptors helps the supervised classification process by means of the proposed machine learning algorithms. The results obtained from the automatic classification (Table 2 and Table 3) of Control 1 vs. the acute group (ACC up to 93.8% for CV and up to 87.5 during FT) and of Control 2 vs. mice with chronic infection (ACC up to 87.5% for CV and up to 66.6% during FT) appear to be competitive with that reported in the state of the art. In [21], using only ECG variables, performances of up to an ACC of 66.7% were obtained during CV and FT for the classification of controls vs. the acute groups. For the classification of control vs. the chronic groups, an ACC of up to 70% was obtained for CV and 100% during FT. In contrast to this work, we present the results of the selection of ECG descriptors and automatic multiclass classification (controls vs. the acute group vs. the chronic group) obtaining an ACC of up to 71.6% for CV and 91.3% for FT. In addition, for this research, we integrated voltage biomarkers, which have hardly been studied in experimental *T. cruzi* infection due to the complexity of acquiring these markers. The additional test with augmented data helped to corroborate the previously obtained performances, showing a consistency between them. Comparing the results shown in Table 4 and Table 5, it can be seen that the RF + FS classifier showed the best overall performance, followed by SVM + FS and ANN + FS. The results obtained suggest that it is possible to detect early infection (the acute phase) and to correctly discriminate between controls, the acute phase of infection, and the chronic phase of infection in a multiclass classification approach, which may be useful in studies of Chagas disease.

## 5. Conclusions

This paper presents the acquisition of ECG signals, statistical comparison, feature selection, and automatic binomial and multiclass classification for the study of experimental infection with *T. cruzi* in a murine model. For this work, we analyzed the acute and chronic phases of infection, with their respective controls, and a multiclass classification scheme (controls vs. the acute group vs. the chronic group). The results showed good results in the early classification of infection (control vs. the acute group), as well as in the automatic classification of control vs. the chronic group. In addition, the results suggest that it is possible to correctly classify controls, mice in the acute infection phase of infection, and mice in the chronic phase of the infection (multiclass classification).

The ECG findings and machine-learning-based classification are useful to distinguish between infected and non-infected animals, regardless of the phase of the infection in this murine model. In the translational context, this work provides information to researchers and clinicians for better understanding the cardiac pathophysiology of *T. cruzi* infection and establishes which ECG changes are important for the diagnosis of *T. cruzi* infection and determining the disease phase.

The P wave voltage and the increased duration of the PR and QTc intervals are some of the most relevant descriptors in the automatic selection of characteristics.

In future work, we intend to incorporate other HRV-derived descriptors, as well as other feature selection and automatic classification strategies, such as a deep architecture. In addition, we intend to incorporate other modalities, such as Doppler or ultrasound, for the study of this infection.

In conclusion, the findings suggest that it is possible to perform correct automatic classification of different phases of *T. cruzi* infection, which can have different applications in experimental and clinical studies of Chagas disease.

## Figures and Tables

**Table 1:** Groups, data proportion, and validation metrics for automatic classification.

Automatic Classification Groups	Day Post Infection Range	Number of Control/Infected Mice	Samples for 4-Fold-CV	Samples for FT	Samples for FT with DA	Validation Metrics
(1) Control 1 vs. Acute	5–35	12/12	8/8	4/4	20/20	ACC AUROC
(2) Control 2 vs. Chronic	60–120	15/15	12/12	3/3	20/20	ACC AUROC
(3) Control 1 and 2 vs. Infected	15–120	27/27	20/20	7/7	40/40	ACC AUROC
(4) Control 1 and 2 vs. Acute vs. Chronic (multiclass)	5–120 Control) 5–35 (Acute) 60–120 (Chronic)	27/12/15	18/8/10	14/4/5	40/20/20	ACC CM

AUROC = Area Under the Receiver Operating Characteristics; CM = Confusion Matrix; CV = Cross-Validation; FT = Final Test; ACC = Accuracy; DA = Data Augmentation.

**Table 2:** Statistical comparisons (median and interquartile ranges) between the mice of Control 1 and the group with acute infection and between the mice of Control 2 and the group with chronic infection, for each ECG descriptor studied. Statistically significant comparisons are shown in bold (* *p* ≤0.05, ** *p*≤0.01).

ECG Descriptor	Control Group 1 Median (Q1–Q3)	Acute Group Median (Q1–Q3)	Control Group 2 Median (Q1–Q3)	Chronic Group Median (Q1–Q3)
HR	446 (387–488)	469 (433–504)	**489** (455–522) *	**454** (408–468) *
P Wave Duration (ms)	10.83 (10.42–12.92)	13.33 (10.94–14.48)	**12.92** (12.08–14.58) **	**18.54** (16.56–21.15) **
P Wave Voltage (mV)	**0.12** (0.11–0.13) **	**0.08** (0.06–0.10) **	**0.13** (0.12–0.15) *	**0.10** (0.07–0.13) *
PR Segment (ms)	**27.92** (23.54–29.69) *	**34.58** (28.65–38.33) *	27.5 (25–31.67)	25.83 (23.23–29.69)
PR Interval (ms)	**39.58** (34.9–41.35) *	**45.21** (41.98–52.29) *	**42.08** (38.75–44.17) *	**45.21** (41.25–47.29) *
Q Wave Voltage (mV)	0.01 (0–0.01)	0.005 (0–0.012)	0.002 (0–0.01)	0.003 (0–0.014)
R Wave Voltage (mV)	0.83 (0.69–0.88)	0.44 (0.28–0.87)	**0.70** (0.59–0.81) *	**0.49** (0.33–0.63) *
S Wave Voltage (mV)	0.11 (0.04–0.21)	0.22 (0.17–0.32)	0.21 (0.14–0.30)	0.20 (0.15–0.24)
QRS Voltage (mV)	0.91 (0.81–1.05)	0.60 (0.52–1.17)	**0.91** (0.76–1.07) *	**0.65** (0.46–0.84) *
QRS Duration (ms)	13.75 (12.6–15)	13.13 (12.50–16.46)	14.58 (12.5–15.42)	13.33 (12.92–15.10)
ST Segment (ms)	4.38 (3.75–8.96)	5.21 (4.27–7.81)	9.17 (8.75–14.17)	8.33 (6.35–13.96)
T Wave Duration (ms)	**37.5** (33.23–44.27) **	**30** (26.67–32.92) **	36.67 (27.92–40.83)	31.25 (15.1–39.27)
T Wave Voltage (mV)	0.05 (0.04–0.05)	0.04 (0.02–0.05)	0.04 (0.03–0.06)	0.04 (0.02–0.05)
QT Interval (ms)	**58.13** (52.71–66.35) **	**49.38** (46.88–52.19) **	58.33 (52.08–66.25)	57.29 (34.79–65.1)
QTc Interval (ms)	**48.80** (47.04–50.88) *	**43.84** (40.55–47.98) *	**54.63** (48.27–59.46) **	**47.41** (31.83–52.29) **
RR Interval (ms)	134.79 (124.06–154.27)	130.83 (118.96–140.94)	**123.75** (115.42–132.08) *	**133.54** (128.96–141.88) *

**Table 3:** Cross-validation results (mean ± standard deviation). The best classification results presented in bold, where all performances are shown as a percentage (%).

Classification Model	Control 1 vs. Acute		Control 2 vs. Chronic		Control 1 and 2 vs. Infected		Control 1 and 2 vs. Acute vs. Chronic
	ACC	AUROC	ACC	AUROC	ACC	AUROC	ACC
RF	81.2 ± 20.7	**100.0 ± 0.0**	87.5 ± 21.3	96.7 ± 6.7	82.5 ± 10.9	88.0 ± 13.3	49.1 ± 13.9
RF + FS	81.2 ± 10.8	**100.0 ± 0.0**	**87.5 ± 13.8**	**100.0 ± 0.0**	**82.5 ± 4.3**	**89.0 ± 8.7**	**71.6 ± 11.7**
ANN	87.5 ± 12.5	80.0 ± 40.0	79.2 ± 13.8	76.7 ± 24.9	80.0 ± 7.1	85.9 ± 6.6	48.2 ± 18.0
ANN + FS	**93.8 ± 10.8**	90.0 ± 20.0	83.3 ± 16.7	96.7 ± 6.7	75.0 ± 11.2	82.0 ± 10.7	65.0 ± 10.2
SVM	**93.8 ± 10.8**	**100.0 ± 0.0**	75.0 ± 25.0	83.3 ± 25.8	75.0 ± 8.7	86.9 ± 4.3	51.3 ± 11.3
SVM + FS	**93.8 ± 10.8**	**100.0 ± 0.0**	87.5 ± 13.8	96.7 ± 6.7	82.5 ± 8.3	85.0 ± 11.4	67.9 ± 10.4

**Table 4:** Final test results with hold-out data. The best classification results are presented in bold, where all performances are shown as a percentage (%).

Classification Model	Control 1 vs. Acute		Control 2 vs. Chronic		Control 1 and 2 vs. Infected		Control 1 and 2 vs. Acute vs. Chronic
	ACC	AUROC	ACC	AUROC	ACC	AUROC	ACC
RF	62.5	86.7	**66.6**	62.5	64.3	78.1	87
RF + FS	**87.5**	**93.3**	**66.6**	**75**	64.3	81.4	**91.3**
ANN	**87.5**	**93.3**	**66.6**	62.5	**71.4**	81.2	87
ANN + FS	**87.5**	**93.3**	**66.6**	**75**	**71.4**	80.1	82.6
SVM	**87.5**	**93.3**	**66.6**	**75**	**71.4**	83.3	78.3
SVM + FS	**87.5**	**93.3**	**66.6**	**75**	**71.4**	**85.4**	87

**Table 5:** Final test results with computationally augmented data. The best classification results are presented in bold, where all performances are shown as a percentage (%).

Classification Model	Control 1 vs. Acute		Control 2 vs. Chronic		Controls 1 and 2 vs. Infected		Controls 1 and 2 vs. Acute vs. Chronic
	ACC	AUROC	ACC	AUROC	ACC	AUROC	ACC
RF	65	84.7	62.5	63.5	63.7	76.4	85
RF + FS	**87.5**	92.1	**67.5**	**74.8**	66.2	**82.9**	**88.7**
ANN	82.5	89.4	60	61.2	62.5	79.6	83.7
ANN + FS	85	91.9	62.5	74	70	78.4	82.5
SVM	80	90.8	65	70.3	70	80.2	78.7
SVM + FS	85	**92.4**	**67.5**	**74.8**	**72.5**	81.6	87.5

## Data Availability

The data used in this research can be shared via email.
